# Annotations

**Published:** 1903-06-27

**Authors:** 


### Annotations.

### The Stage Child.

Prominent actors are protesting against the "Employment of Children" Bill in so far as it applies to children employed at theatres. Mr. Forbes Robertson accuses a number of M.P.'s of "wilful and therefore criminal ignorance of theatrical life," and sets down to mere ignorance the opposition of these gentlemen to the employment of children on the stage. Assuredly the ignorance which condemns without investigating is blameworthy, but does it need a special knowledge of the theatre to see reasons why the stage is not a place for young children? We are not speaking of the supposed moral dangers, concerning which a great deal of nonsense is talked. The children in all probability run no greater moral risks there than they would at home. But the physical objections are strong enough. Children ought to go to bed at sundown, and pass as much of their time as possible in fresh air. Stage children are kept up until nearly midnight in a heated and usually vitiated atmosphere. This cannot be good for their health. Speaking parts for children are commonest in "domestic" dramas of a strained emotionalism, and the clever children who play these parts must, to do them well—as they often do—be strung up to a degree of nervous tension which one can hardly consider good for them. We are often assured by those who favour the employment of children in the theatre, that at the training schools for dancing or singing where the children are prepared, ample provision is made for their education. The children in all probability will be tired, and the teachers languid. And when children are actually employed on the stage every evening, they cannot be fit to gain much by lessons; indeed, all their spare time would need to be given to rest and play. These considerations are engaging the attention of Parliament and its final decision is awaited with interest.

### Walking as an Exercise.

Those who thought that cycling was bound to interfere with the tramping powers of the present generation must have been agreeably surprised at the large numbers who turned out lately for walking matches at high speed. It is not an accomplishment that is ever likely to decline. We must all walk, just as we must all talk prose. It is said that there are persons who make it a rule never to walk when they can ride. But it is never good economy to spare shoe leather. The man who keeps his legs in condition (it is equally true of the female) is doing much to keep his nerves and his brain in condition; and he will probably be able to measure his degree of vigour by observing the strength of his knees. The great distances that have to be traversed in such a place as London, often under quite favourable conditions of clean and broad pavements, are a necessary regimen for many, which is altogether good for them. It is true that walking is not the ideal exercise, from the anatomical point of view. It is better than cycling, for it allows of that half-swing of the pelvis at the advance of the legs alternately, which is wanting in the cyclist's seat; and it gives much better play to the arch of the foot and to the ankle. "I ken the manner of his gait, he rises on the toe," is the distinction of Diomed, in "Troilus and Cressida;" but that is what every fair heel-and-toe walker should do, whatever Shakespeare may have meant specially in his gallant Greek. The chief demerit of pedestrianism is, that it gives no exercise for the arms and shoulders, and not much for the loins. The kind of walking which fulfils every end of exercise is that which one takes, with a golf-club in hand, round a links. The swing of the body in wielding the club is a variety of muscular exertion which is too seldom practised, and cannot be too much prized. A golfer will sometimes have remarked that certain groups of his muscles must be comparatively unexercised in ordinary, from the stiffness which he feels in them after his first day's play following a long interval; and among them are the muscles of the loins and flanks. It may be said to be a sound principle, needing no proof, that the better the condition of the muscular walls, the safer the state of the organs contained within them.

### Municipal Lodging-houses.

It is contended not only that municipalities should erect lodging-houses for the poorest workers, and for casual wayfarers, but that the municipalities should have a monopoly of such places, as the only way of preventing their becoming centres for the dissemination of infection. The lodgers would, on their arrival, be washed by the attendants and then examined by some competent person—perhaps a medical man appointed by the municipality for such duty—so that any symptoms of infectious disease might be noted at once, and the sufferer placed, we presume, in a sort of isolation ward, where he could be under observation until it was clear whether or not he should be removed to the hospital. Having regard to what we know of the dissemination of infection by tramps, it is obvious that such a plan has many advantages if it can be carried out. Having regard to what we know of tramps and their ways, we doubt the possibility of its success. The tramp is a rebel against authority; that, rather than misfortune, is usually at the foundation of his tramping. Probably he will not wash at all; certainly he will not submit to be washed by the authorities. Either he will spend perhaps twice as much as his bed in the municipal lodging-house would cost on far inferior accommodation in a private house, or, if poverty drive him, he will deposit his takings with some friend or tramps' banker, and take his wash and medical inspection gratuitously at a casual ward. It may be said that the municipal monopoly—without which the whole plan is futile—would prevent the first possibility, but in all probability it would only develop a number of unlicensed lodging-houses, which the police would have to hunt out as they now have to do with illicit drinking-shops. Moreover, the tramp's clothes, as well as his person, are likely to be vehicles for the conveyance of infection. Supposing these are to be disinfected every time he goes to a new abode, their wearing qualities, such as they are, would be so seriously affected that he might reasonably put in a plea for compensation. The scheme altogether, although admirable in theory, is full of difficulties, quite apart from the very obvious one of its expense.

